# Risk factors for lymph node metastasis in early gastric cancer patients: Report from Eastern Europe country– Lithuania

**DOI:** 10.1186/s12893-017-0304-0

**Published:** 2017-11-23

**Authors:** Rimantas Bausys, Augustinas Bausys, Indre Vysniauskaite, Kazimieras Maneikis, Dalius Klimas, Martynas Luksta, Kestutis Strupas, Eugenijus Stratilatovas

**Affiliations:** 1 Department of Abdominal Surgery and Oncology, National Cancer Institute, Santariskiu str. 1, Vilnius, 08660 Lithuania; 2 Faculty of Medicine, Vilnius University, Ciurlionio str. 21, Vilnius, 03101 Lithuania; 3 School of Medicine, Georgetown University, Washington, D.C. USA; 4 Center of Abdominal surgery, Vilnius University Hospital Santaros Klinikos , Santariskiu str. 2, Washington, 08661 USA

**Keywords:** Early gastric cancer, Lymph node metastasis, Risk factors, Mucosal tumor, T1a

## Abstract

**Background:**

Current risk factors for lymph node metastasis in early gastric cancer have been primarily determined in Asian countries; however their applicability to Western nations is under discussion. The aim of our study was to identify risk factors associated with lymph node metastasis in Western cohort patients from the Eastern European country - Lithuania.

**Methods:**

A total of 218 patients who underwent open gastrectomy for early gastric cancer were included in this retrospective study. After histolopathological examination, risk factors for lymph node metastasis were evaluated. Overall survival was evaluated and factors associated with long-term outcomes were analyzed.

**Results:**

Lymph node metastases were present in 19.7% of early gastric cancer cases. The rates were 5/99 (4.95%) for pT1a tumors and 38/119 (31.9%) for pT1b tumors. Submucosal tumor invasion, lymphovascular invasion, and high grade tumor differentiation were identified as independent risk factors for lymph node metastasis. Submucosal tumor invasion and lymphovascular invasion were also associated with worse 5-year survival results.

**Conclusion:**

Our study established submucosal tumor invasion, lymphovascular invasion, and high grade tumor differentiation as risk factors for lymph node metastasis.

## Background

Gastric cancer is one of the leading causes of cancer death worldwide. It is believed that early detection and appropriate treatment can reduce mortality caused by gastric cancer. After the detection of cancer, preoperative disease staging must be performed to plan an ideal treatment for each individual patient. Next, the absence or presence of lymph node metastasis (LNM) must be confirmed to apply the treatment strategy. Lymph node status has great significance to the path of care chosen in early gastric cancer (EGC). It is not only important for proper treatment, but also for the prognosis of survival [1, 2]. Additionally, confirmation of LNM is crucial when an endoscopic approach is considered, because such a procedure does not cure the disease in lymph nodes. One obstacle in determining lymph node status has been the uncertainty of radiological tests. Staging of gastric cancer typically utilizes a variety of imaging modalities, such as computed tomography (CT), magnetic resonance imaging (MRI), endoscopic ultrasounds and combined positron tomography, as well as, laparoscopic staging and cytogenetic analysis of peritoneal fluid in appropriate patients [3, 4]. The evaluation of metastatic infiltration of lymph nodes is mostly based on CT and MRI imaging. However, neither have the correct high sensitivity and specificity for the detection of LNM in gastric cancer [5, 6]. The lack of accurate radiological imaging calls for research of risk factors for LNM in EGC, which are used when endoscopic treatments of EGC are considered. According to the European Society for Medical Oncology (ESMO) guidelines, gastric adenocarcinomas staged as T1a, well-differentiated, less than 2 cm diameter and not ulcerated have very low or no risks for LNM. Those that fall under such standard criteria are eligible for endoscopic mucosal resection (EMR)/endoscopic submucosal dissection (ESD) [7–10]. These guidelines are based on known risk factors for LNM, which have been determined in Asia. However, recently published data has revealed race as an independent risk factor for LNM, raising concern whether such risk factors can be considered in Western countries [11, 12].

The aim of our study was to integrate our experience of treating early gastric cancer with open gastrectomy to identify risk factors for LNM in EGC in Western populations.

## Methods

This retrospective study included 218 patients who underwent surgical treatment for EGC in the Department of General and Abdominal Surgery and Oncology, National Cancer Institute, Vilnius, Lithuania between January 2005 and December 2015. In total, 1654 patients with gastric adenocarcinoma were operated on at the institution during the study period. 1436 (86,8%) patients underwent surgery for advanced gastric cancer and 218 (13,2%) for EGC.EGC was defined as a cancer that does not invade past the submucosa, irrespective of regional lymph node metastasis (T1 any N). None of the patients received neoadjuvant chemotherapy or radiotherapy prior to surgery. All patients had morphological gastric cancer verification before surgery, except in a few cases when it was not possible due to technical feasibility. Depending on cancer localization in the stomach and histological characteristics of the tumor, the type of surgery - total or subtotal gastrectomy- was determined before the operation. Reconstruction after a total gastrectomy was performed with esophagojejunostomy using a jejunal loop and Braun’s side-to-side enteroanastomosis (m.Omega). Reconstruction after subtotal gastrectomy consisted of an antecolic end-to-side gastrojejunostomy with Braun’s jejunojejunostomy (m.Balfur).The standard lymphanodectomy in our institution was a D2 lymph node dissection and was performed in accordance with the guidelines of the Japanese Research Society for Gastric Cancer. D1 lymphanodectomy was an alternative option based on the surgeon’s individual decision. R0 resection was defined as no tumor remaining macroscopically and microscopically, and was achieved in all cases. Specimens’ histological examination was performed in the National Center of Pathology, Vilnius, Lithuania. Standard histological examination protocol included entire lesion examination with 5 mm wide slices. All of the dissected lymph nodes were analyzed, each lymph node was embedded in paraffin, and at least two sections were prepared and visualized. Immunohistochemistry was performed using an anti-podoplanin antibody (D2–40) and CD34 antibody to identify and distinguish the lymphatic endothelium. The rate of LNM was calculated after histological evaluation. Various clinicopathological parameters such as gender, age, primary tumor invasion, tumor differentiation grade, lymphatic and vascular invasion, tumor type according to Lauren classification, ulceration, tumor size and localization were evaluated as possible risk factors for LNM. Analysis of postoperative morbidity and intra-hospital 30- and 90-day mortality rates were performed. Surgical complications were classified by Clavien-Dindo classification.

Outcomes of interest included the overall survival (OS) rates. OS was defined as the duration from the date of surgery to the date of death. Data on survival and death were obtained from Lithuania’s Cancer register and Lithuania’s death register. The date of the last follow up was 31 December 2016. 6 (2.7%) patients were lost during the follow-up period. Mean and median follow-up periods were 68 and 63 months (range from 0 to 142) respectively.

### Statistical analysis

All statistical analyses were conducted using the statistical program SPSS 16.0 (SPSS, Chicago, IL, USA). Clinicopathological characteristics were analyzed by a 2-tailed *t* test, one-way ANOVA test, Chi-square test, or Fisher exact test. The risk factors found to be significant in univariate analysis were included in subsequent multivariate logistic regression analyses to identify the independent variables associated with lymph node metastasis in patients with gastric cancer. Overall survival was analyzed by the Kaplan–Meier method, and the curves drawn were compared by the log-rank test. Multivariate survival analysis was performed using the Cox proportional-hazards model (hazard ratio and 95% confidence intervals). In all statistical analyses, a *p* value of <0.05 was considered to be significant.

## Results

From January 2005 to December 2015, 218 patients undergoing total or subtotal gastrectomy for EGC were included in this study. All of the patients were of Caucasoid race. Baseline characteristics of all patients are shown in Table 1.

**Table 1:** Baseline characteristics of all patients

Variable		
Age (mean ± SD, range) (min.-max. Years)		65.58 ± 12.33 (27–88)
BMI (mean ± SD, range) (kg/m^2^)		26.31 ± 5.41
Count of retrieved lymph nodes (mean ± SD, range) (min.-max.)		19.89 ± 9.69 (3–70)
Gender	Male	117 (53.7%)
	Female	101 (46.3%)
ASA score	I	23 (10.6%)
	II	105 (48.2%)
	III	87 (39.9%)
	IV	3 (1.4%)
Tumor localization	Lower third	79 (36.2%)
	Middle third	125 (57.3%)
	Upper third	14 (6.4%)
Tumor invasion	Mucosal	99 (45.4%)
	Sub-mucosal	119 (54.6%)
Lymph node status	Positive	43 (19.7%)
	Negative	175 (80.3%)
Tumor differentiation grade	G1	44 (20.2%)
	G2	70 (32.1%)
	G3	104 (47.7%)
Type of surgery	Total gastrectomy	38 (17.4%)
	Subtotal gastrectomy	180 (82.6%)
Type of lymphanodectomy	D1	23 (10.6%)
	D2	195 (89.4%)

There were 117 (53.7%) men and 101 (46.3%) women, with a mean age of 65.58 ± 12.33 years. Total gastrectomy was performed in 38 cases and subtotal gastrectomy in 180 cases. Forty-five of 220 patients had postoperative complications, with four of them lethal. Postoperative mortality and morbidity rates were 1.8 and 20.6% respectively. The vast majority of complications (29 of 45, 64.4%) were not life threatening and did not require any surgical, endoscopic, or radiological interventions. According to Clavien-Dindo classification they were either grade I or II complications. Grade III complications occurred in 8 cases (3.6%), and reoperation was indicated for 5 (2.3%) patients. Complications requiring intensive care unit management (grade IV) were rare – 4 cases (1.8%). Four patients (1.8%) died during the intra-hospital period after postoperative complications had occurred. Causes of death for these patients were as follows. One patient had anastomotic leakage and peritonitis, while three patients died from non-surgical complications: pulmonary embolism – 1 case, pneumonia and sepsis - 1 case and acute cardiovascular insufficiency – 1 case. Mean hospitalization time was 17.34 ± 5.90 days and mean postoperative period was 13.00 ± 5.39 days. 30 day mortality rates were higher when compared to intra-hospital mortality rates and involved 6 (2,8%) cases. Three additional deaths were registered between the 31st and 90th postoperative day. 90 day postoperative mortality rates reached 4.1%. Higher 90 days mortality rates were associated with elderly age (≥75 years; 12.5% vs 1.2%, *p* = 0.010). Other factors such as gender, smoking status, obesity (BMI > 30), ASA score, extent of lymphanodectomy, type of surgery, and tumor localization did not impact mortality rate, *p* > 0.05.

Majority of the patients underwent a D2 lymphadenectomy – 195 (89.4%). The average number of removed lymph nodes was 19.89 ± 9.69. After performing histological examination of operative material, LNM were revealed in 43 (19.7%) cases. Factors associated with LNM were evaluated by univariate analysis. There was a significantly higher risk for LNM in tumors with submucosal layer infiltration (compared to mucosal infiltration, *p* = 0.001), lymphovascular invasion (LV+ vs LV-, *p* = 0.001), high grade differentiation (G3 vs G1&G2, *p* = 0.047), diffuse or mix type according to Lauren classification (compared to intestinal type, *p* = 0.012), and diameter exceeding 2 cm (compared to tumors ≤ 2 cm, *p* = 0.026). Age, gender, tumor localization, ulceration, and signet ring cell carcinoma had no significance in the presence of LNM (Table 2).

**Table 2:** Clinicopathological data of patients with EGC and univariate analysis of risk factors for lymph node metastasis

		LNM-	LNM+	*p*	Odds ratio (95% CI)
Gender	Male	99 (84.6%)	18 (15.4%)	*p* = 0.090	1.80 (0.92–3.55)
	Female	76 (75.2%)	25 (24.8%)		
Age		65.26 ± 12.17	66.91 ± 13.03	*p* = 0.433	–
Tumor localization	Lower 1/3	62 (78.5%)	17 (21.5%)	*p* = 0.457	–
	Middle1/3	100 (80.0%)	25 (20.0%)		
	Upper 1/3	13 (92.9%)	1 (7.1%)		
Tumor invasion	T1a	94 (94.9%)	5 (5.1%)	*p* = 0.001	8.82 (3.31–23.46)
	T1b	81 (68.1%)	38 (31.9%)		
Tumor differentiation	G1 & G2	100 (87.7%)	14 (12.3%)	*p* = 0.006	2.76 (1.36–8.57)
	G3	75 (72.1%)	29 (22.4%)		
Lymphovascular invasion	LV+	12 (40%)	18 (60%)	*p* = 0.001	9.78 (4.20–22.72)
	LV-	163 (86.7%)	25 (13.3%)		
Lauren classification	Diffuse & mix	59 (71.1%)	24 (28.9%)	*p* = 0.012	2.09 (1.20–3.64)
	Intestinal	106 (86.2%)	17 (13.8%)		
Tumor size	≤2 cm	91 (86.7%)	14 (13.3%)	*p* = 0.026	2.27 (1.12–4.59)
	>2 cm	83 (74.1%)	29 (25.9%)		
Ulceration	Ulcerated	58 (74.4%)	20 (25.6%)	*p* = 0.114	1.73 (0.88–3.42)
	Non-ulcerated	116 (83.5%	23 (16.5%)		
Signet ring cell	Yes	11 (73.3%)	4 (26.7%)	*p* = 0.513	1.46 (0.44–4.86)
	No	149 (80.1%)	37 (19.9%)		

The multivariate analysis showed that submucosal tumor invasion, lymphovascular invasion, and high tumor differentiation grade were independent risk factors for lymph node metastasis (Table 3).

**Table 3:** Multivariate analysis of risk factors for lymph node metastasis

Factor	*p* value	Odds Ratio (95% CI)
Submucosal tumor invasion (T1b)	*p* = 0.001	6.55 (2.28–18.81)
Tumor differentiation grade G3	*p* = 0.045	2.01 (1.03–14.66)
Lymphovascular invasion	*p* = 0.001	6.06 (2.28–16.07)
Tumor size >2 cm	*p* = 0.155	1.82 (0.79–4.19)
Diffuse type according to Lauren classification	*p* = 0.693	1.29 (0.35–4.69)

### Survival analysis

**Fig. 1:**
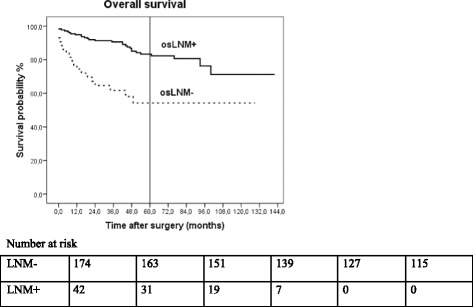
Five-year overall survival rate of patients with EGC according to lymph node status

**Fig. 2:**
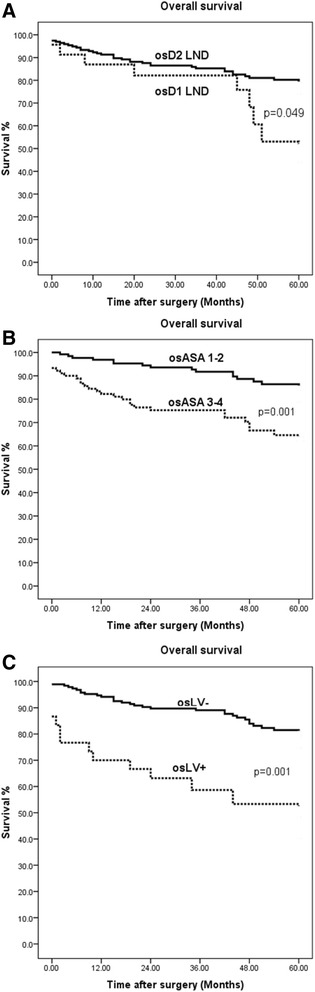
Five-year overall survival rate of patients with EGC according to lymphanodectomy (**a**), ASA score (**b**) and lymphovascular invasion (**c**)

5-year overall survival was 83.3% in patients without LNM and 54.2% in patients with LNM, *p* = 0.001 (Fig. 1). In the univariate analysis, LNM (p = 0.001), higher ASA classification (ASA III/IV p = 0.001), D1 lymphanodectomy (*p* = 0.049), and lymphovascular invasion (p = 0.001) had a negative effect on 5-year survival (Fig. 2). In multivariate analysis, lymphovascular invasion (*p* = 0.028; HR 2.19; 95% CI 1.08–4.42) and age (*p* = 0.020; HR 1.04; 95% CI 1.01–1.08) were discovered as independent factors with negative influences on the postoperative overall survival rate.

## Discussion

The definition of EGC was established by the Japanese Gastroenterological Endoscopic Society in 1962, originally characterizing EGC as gastric cancer that invades no deeper than the submucosa regardless of lymph node metastasis. EGC is more commonly diagnosed in Asia compared with Western countries. In Japan, EGC comprises approximately 60% of all diagnosed gastric cancers, whereas in Western countries the incidence of EGC varies from 10% to 20%. Such differences could be explained by the presence of more screening programs in Asian countries and also by different interpretations of histological changes. Western pathologists consider invasion into the lamina propria of the mucosa mandatory characteristics for the diagnosis of carcinoma, whereas nuclear and structural features are more important in Japan. Therefore, EGC lesions diagnosed in Japan are potentially diagnosed as high grade dysplasia in Western countries. This distinction could partly explain the higher incidence of EGC in the Asian population and be responsible for better patient prognoses when compared to Western counterparts [11]. Several studies in Asian countries have declared excellent 5-year survival rates for EGC patients with overall survival exceeding 90%. Comparatively, our study presents worse results with the 5-year overall survival rate reaching only 77,6%. Since there is a disparity in histological interpretations and a difference in average life expectancy amongst distinct regions, it is difficult to compare data of several studies using the same statistical indicators [2, 13]. Disagreement between Asian and Western interpretations of pre-cancerous lesions and EGC could also influence results of studies analyzing risk factors and rate of LNM in EGC patients. During the last 5 years, 17 studies investigating factors associated with LNM in EGC were published. Eleven studies came from Asian countries and six from Western countries (Table 4).

**Table 4:** Lymph node metastasis in early gastric cancer – literature review and our results

Author	Country	Year	No. of patients		LNM+ in T1a cancer patients	LNM+ in T1b cancer patients	Risk factors for LNM
Studies from Asian countries							
Lim MS. et al. [14]	South Korea	2011	376		2.8%	18.4%	T1a: tumor size > 2 cm and lymphovascular invasion T1b: macroscopic type (elevated) and lymphovascular invasion
Ren G. el al. [25]	China	2013	202		9.0%	22.5%	Depth of invasion
Wang L. et al. [26]	China	2013	242		5.5%	20.0%	Depth of invasion, lymphovascular invasion.
Nakagawa M. et al. [27]	South Korea	2015	1042		Not available	Not available	Depth of invasion, tumor size, ulceration, age and positive nodal status by CT.
Wang Y. [16]	China	2015	198		6.0%	56.2%	Depth of invasion. Tumor size. Ulceration. histological type and venous invasion.
Park JH. et al. [28]	South Korea	2015	2270		2.8%	19.0%	Depth of invasion, tumor size >3 cm and lymphovascular invasion
Fang WL. et al. [29]	Taiwan	2015	391		4.9%	21.4%	T1a: Lauren’s diffuse type and lymphatic invasion T1b: lymphatic invasion
Zhao LY. et al. [15]	China	2016	687		15.5%	35.9%	Depth of invasion. tumor size > 2 cm, ulceration, lymphovascular invasion, differentiation
Wang YW. et al. [30]	China	2016	230		8.5%	28.6%	Depth of invasion, tumor size ≥ 2 cm and P53 overexpression
Sekiguchi M. et al. [19]	Japan	2016	3131		4.2%	20.2%	Depth of invasion, tumor size ≥ 2 cm, ulceration, lymphovascular invasion, differentiation
Zheng Z. et al. [17]	China	2016	597		3.0%	18.3%	Depth of invasion, ulceration, lymphovascular invasion, age, differentiation.
Studies from Western countries							
Milhomem LM. et al. [31]	Brazil	2012	126	7.8%	22.6%	Depth of invasion, tumor size > 5 cm, ulceration and lymphatic invasion.	
Bravo Neto GP. et al. [23]	Brazil	2014	26	16.7%	42.9%	Not available	
Fukuhara S. et al. [10]	USA	2014	104	7.1%	35.4%	Lymphovascular invasion, non-Asian race and younger age.	
Haist T. et al. [22]	Germany	2016	124	1.9%	22.5%	Depth of invasion, lymphovascular invasion.	
Ahmad R. et al. [18]	USA	2016	67	4.3%	31.8%	Lymphovascular invasion and positive nodal status by endoscopic ultrasound.	
Ronellenfitsch U. et al. [24]	Germany	2016	275	3.9%	18.2%	Depth of invasion, lymphovascular invasion, diffuse- and mixed-type according to Lauren.	
Our study	Lithuania	2017	218	5.1%	31.9%	Depth of invasion, lymphovascular invasion and tumor differentiation grade	
Indication for endoscopic treatment of EGC according to different guidelines							
ESMO-ESSO-ESTRO	Well-differentiated, lesion is ≤2 cm in diameter, confined to the mucosa and not ulcerated.						
NCCN	Well or moderately well differentiated, lesion is ≤2 cm in diameter, confined to the mucosa, does not exhibit lymphovascular invasion						

Rates of LNM reported in various Asian and Western countries were varying. In Asia LNM rates ranged from 2.8% to 15.5% for patients with tumors invading only the mucosal layer [14, 15] and from 18.3% to 56.2% for patients with tumors invading the submucosal layer [16, 17]. Respectively, in Western countries, rates of LNM varied from 1.9% to 16.7% when the tumor was localized to the mucosa and from 18.2% to 42.9% when the tumor invaded the submucosal layer [17, 18]. Our study results were similar; rates of LNM for patients with T1a and T1b cancer were 5.1 and 22.4%, respectively. Risk factors for LNM determined in various studies were also differing. In Asian studies, the most frequently mentioned factors were depth of invasion (9 of 11 studies), tumor size (7 of 11 studies), and lymphatic or lymphovascular invasion (7 of 11 studies). In Western studies, lymphovascular invasion has been of recent focus in five of six studies with our results also confirming lymphovascular invasion as a risk factor for LNM. Another risk factor which was studied in our data, submucosal tumor invasion, was mentioned in 3 of 6 previously published Western studies. Tumor differentiation was only mentioned as a risk factor for LMN in reports from Asian nations [15, 17, 19]. To our best knowledge, our study is the first report of Western countries which confirms tumor differentiation as an independent risk factor for LNM. Despite the discussed variation between Asian and Western regions, the use of EMR/ESD as a treatment option for ECG is increasing in the West [8]. Western guidelines such as the National Comprehensive Cancer Network (NCCN) guidelines for gastric cancer treatment and ESMO clinical practice guidelines recommend the endoscopic approach as an appropriate option for some cases of intramucosal gastric cancer. Indications for endoscopic treatment reported by these guidelines are very similar but display several discrepancies. While both guidelines note that tumor diameter should not exceed 2 cm, only the NCCN guidelines indicate lymphovascular invasion as a required criteria. On the other hand, the ESMO guidelines are more strict on the limits for differentiation grade. They suggest that only well differentiated tumors should be treated endoscopically. NCCN guidelines are more liberal by indicating that both well and moderately-well differentiated tumors can be treated by the endoscopic approach. Additionally, ESMO guidelines limit indications with ulceration criteria and NCCN guidelines do not. In our cohort of patients, 30 (13.7%) of 218 patients would have met the criteria for endoscopical treatment according to NCCN guidelines. 1 (3.13%) of these 30 patients had histologically confirmed LNM. Fewer patients fit ESMO criteria (13 of 218 patients) and none of them had LNM. While LNM risk is low or equal to zero for patients who match standard endoscopical treatment criteria, implementation of expanded criteria in Western countries has been questioned. Furthermore, suspicions about different tumor behavior have increased after Ikoma et al. and Fukuhara et al. studies recently published that race is a risk factor for lymph node metastasis in gastric cancer [11, 12].

On the other hand, even non-curative endoscopic treatment could lead to satisfactory results. Hatta et al. recently published a large multi-center study, in which they evaluated and compared long term outcomes for patients who underwent non-curative endoscopic treatment of EGC followed by either radical surgery or only follow-up. The study revealed that patients who underwent radical surgery had significantly longer 3- and 5-year overall survival (OS) and disease-specific survival rates (DSS). However, the difference in DSS rates was rather small (99.4% vs. 98.7%) compared to the difference in OS rates (96.7% vs. 84.0%). Estimated rates of recurrence were significantly different, although both were low; 1.3% in the radical surgery group and 3.1% in the follow-up group. Nonetheless, positive results according to DSS and recurrence rates in the follow-up group should be interpreted carefully due to different clinicopathological backgrounds between the two groups. Some risk factors for LNM (lymphatic invasion or deeper submucosal invasion) were significantly more frequent in the radical surgery group [20]. Furthermore, Nakamura et al. provided a direct correlation between lymphatic infiltration and worse survival [21]. Up to this date, evidence of a correlation between lymphatic invasion and worse survival results have been demonstrated only by studies performed in Asia, with Western studies confirming these findings. Haist et al. analyzed lymphatic invasion and survival result correlation in a Western cohort, however they did not display a significant effect of lymphatic invasion on longtime survival results [22]. Consequently, our study became the first of such Western reports to present lymphovascular invasion as an independent prognostic factor associated with worse long-term survival results.

Several limitations of our study must be taken into consideration. First, it is a retrospective study originating from a single centre conducted over a long period of time. Second, surgical techniques used were not standardized. Even though the D2 lympahadenectomy remained institutionally standard over the entire study period and the number of examined lymph nodes was sufficient, the extent of resection and confirmation of lymph node removal was not clear in every case. Appropriate lymphadenectomy is important because the risk of lymph node metastasis could be underestimated in cases with incomplete lymphanodectomy. However, we believe that the quality of th obtained histological specimens was accurate and adequate, while the average number of removed lymph nodes was 19.89 ± 9.69. More than 15 lymph nodes were removed for 72% of the patients and only 9% of cases included less than ten dissected lymph nodes. Also, patients with insufficient lymphanodectomy were not excluded from our study to avoid discrepancies as we compared our study results to five other studies from Western countries, where patients with less than 15 examined lymph nodes were included [18, 20, 22–24]. Moreover if only patients with ≥15 resected LN would have been analyzed, the percentage of lymph node metastasis would not be much higher than in the whole series (T1a cancer - 5,1% vs 5,6%, T1b cancer 31,9% vs 36%). Therefore, the entire continuous series was used to avoid selection bias. Third, we were unable to use follow-up records, preventing us from estimating and evaluating recurrence rates, disease free survival and disease specific survival. Fourth, although our study was large enough when compared to other similar Western studies, the absolute number of patients with LNM was still relatively low. This reduces the statistical power of our analyses and the confidence of identifying correct risk factors for LNM.

## Conclusion

In our study, LNM occurred in 19,7% of EGC cases. However, depending on varying criteria of several distinct guidelines, the rate of LNM meeting indications for an endoscopic resection was low or equal to zero. Additionally, this study identified submucosal tumor invasion, lympovascular invasion, and high grade tumor differentiation as risk factors for lymph node metastasis. Lymphatic invasion and submucosal tumor invasion were associated with worse 5-year overall survival results. Endoscopical treatment of EGC should be performed within the standard criteria. If risk factors for LNM are present in histological specimens, surgery with adequate lymphadenectomy should be followed. To successfully utilize the endoscopical approach in Western countries, criteria need to be expanded and applied to the appropriate population. For safe implementation, further research should be conducted by Western studies.
